# Critical Appraisal of International Guidelines for the Management of Diabetic Neuropathy: Is There Global Agreement in the Internet Era?

**DOI:** 10.1155/2015/519032

**Published:** 2015-04-27

**Authors:** Mingfang Sun, Min Zhang, Jing Shen, Juping Yan, Bo Zhou

**Affiliations:** Department of Endocrinology, The First Affiliated Hospital of Chongqing Medical University, Chongqing 400016, China

## Abstract

*Purpose.* The management of diabetic neuropathy (DN) can be challenging. There exist many guidelines for DN management, but the quality of these guidelines has not been systematically evaluated or compared. The objective of our study was to assess the quality of these guidelines as a step toward their future optimization, the development of international guidelines, and, ultimately, the improvement of the care process. *Methods.* Relevant data were selected to identify international guidelines. The Appraisal of Guidelines for Research and Evaluation II (AGREE II) tool was used to evaluate the quality of the selected guidelines. In addition, the reviewers summarized and compared all of the recommendations from the included guidelines for DN's management. *Results.* Thirteen guidelines were included after the selection process. According to AGREE II, few guidelines scored well for all three aspects of DN management. Detailed comparisons revealed that these guidelines provide inconsistent recommendations, making it difficult for diabetes clinicians to choose appropriate guideline. *Conclusions.* The quality of most guidelines for the management of DN should be improved. Further studies should concentrate on developing internationally accepted and evidence-based guidelines that could be used for clinical decision making to improve patient care.

## 1. Introduction

Diabetic neuropathy (DN) is a neuropathic disorder characterized by diabetes mellitus (DM) with peripheral neuropathy lacking other possible causes. DN, one of the most common long-term complications of DM, affects nearly 50% of patients with diabetes [1, 2]. DN is an important cause of morbidity and mortality and is associated with a tremendous financial burden in China and the rest of the world [3, 4]. Neuropathic disorders involve numerous abnormalities that affect proximal and distal peripheral sensory and motor nerves as well as the autonomic nervous system; thus, the clinical manifestations of DN vary widely. Some cases are silent and go undetected, whereas other cases may involve a wide variety of symptoms, including intense neuropathic pain, paresthesia, and gastrointestinal, bladder, and sexual dysfunction [5]. Because these nonspecific, insidious symptoms and signs are often observed in many other diseases, the management of DN can be challenging. As a consequence, over the past decade, several national and international organizations have published guidelines for the care of patients with DN to enable clinicians to make appropriate treatment decisions.

Clinical practice guidelines (CPGs) are developed systematically to help practitioners and patients choose the appropriate care for specific clinical situations. Indeed, it has been demonstrated that the proper use of high-quality guidelines can improve patient outcome [6, 7]; thus, the quality of the guidelines is considerably important. CPGs are also thought to be globally valid because the evidence upon which these guidelines are based is universally available, and the key recommendations should accordingly be similar throughout the world. However, our studies and those of others have demonstrated that guidelines addressing the same clinical circumstances that are developed by different organizations are occasionally inconsistent [8, 9]. In the modern era, Internet-based dissemination is thought to increase the accessibility of CPGs. Although various guidelines are easily obtained online, in certain cases of inconsistencies in the recommendations from different guidelines, it is difficult for practitioners to choose the appropriate recommendations. It has been demonstrated that when a guideline is poorly presented or provides recommendations that conflict with other guidelines, thus making it difficult to choose the appropriate course of action, clinicians and others involved in patient care might not follow any guidelines at all [10].

To the best of our knowledge, no published appraisals of the quality of contemporary guidelines for the treatment of DN are available online. The purpose of this study was to systematically retrieve all existing guidelines regarding the treatment of DN and to assess and compare their quality using Appraisal of Guidelines for Research and Evaluation II (AGREE II) instrument and to compare the recommendations given by different guidelines, with the goal of helping diabetes clinicians working in busy clinical practices choose the most appropriate guidelines and recommendations.

## 2. Materials and Methods

### 2.1. Data Sources and Literature Selection Process

A systematic search was performed to retrieve relevant guidelines regarding the management of DN. Guidelines were identified using computer searches of the Guidelines International Network Library, the National Institute for Health and Clinical Excellence (NICE) database, the Medline database, and the National Guidelines Clearinghouse. Additional guidelines were obtained by reviewing the homepages of international medical institutions and other relevant websites. The searches were conducted using the following controlled terms: diabetic neuropathy, diabetic peripheral neuropathy, and painful neuropathy. Additionally, we used terms corresponding to the following internationally agreed-upon classifications [11]: (1) for DN: diabetic distal symmetric polyneuropathy, sensory neuropathy, sensorimotor neuropathy, and autonomic neuropathy; (2) for focal and multifocal DN: cranial neuropathy, truncal neuropathy, focal limb neuropathy, proximal motor neuropathy, and chronic inflammatory demyelinating polyneuropathy. All searches were limited by the term “guideline.”

We developed a series of inclusion criteria to select guidelines: (1) the target group included patients with DN; (2) the guidelines focused on the management of DN; (3) the full text was available online; (4) the guidelines were written in English; and (5) the guidelines were published between June 1, 2004, and June, 2014.

Guidelines were excluded according to the following criteria: (1) did not meet the definition of a guideline or satisfy the definition of a narrative review; (2) focused on the treatment of DM but did not address DN in detail; or (3) offered a review of DN but did not present specific recommendations. Furthermore, if different versions of the guidelines were available, only the latest edition was selected.

### 2.2. Comparison of the Recommendations in the Guidelines

The successful treatment of DN must include three key elements: (1) the implementation of strategies to minimize risk factors and complications, (2) management based on the pathogenetic mechanisms of DN, and (3) the use of therapies to relieve symptoms [12, 13]. Therefore, two reviewers individually collected and summarized all of the recommendations from the included guidelines for these three independent aspects of DN treatment. The clarity and the completeness of each summary were assessed by the other reviewer, and any disagreements were resolved by consensus or by a third reviewer.

### 2.3. Quality Assessment

We evaluated the selected guidelines with respect to the three independent aspects of DN treatment utilizing Appraisal of Guidelines for Research and Evaluation II (AGREE II) instrument (2013 version). The AGREE II tool is a validated assessment instrument that is designed to provide a framework for the evaluation and monitoring of clinical guidelines [14], and it can be used to measure and quantify guideline quality. AGREE II instrument consists of 23 items within 6 individual domains: (1) scope and purpose; (2) stakeholder involvement; (3) rigor of development; (4) clarity of presentation; (5) applicability; and (6) editorial independence. Each domain was rated individually by three reviewers (all diabetologists) who were blinded to one another's ratings, and each item focused on one key independent aspect of guideline quality. Each item within an individual domain was rated from 1 (strongly disagree) to 7 (strongly agree) by the three reviewers. A score of 1 was given when little or no relevant information was presented. Scores from 2 to 6 were given when the statements did not fully meet the criteria or consider one item in the criteria, and the scores increased as the criteria were more fully met or greater consideration was provided. A score of 7 was given when the statement met all criteria or fully considered its standards. All items with a score difference of more than 2 or 3 between reviewers were discussed further. The detailed criteria for each item are available in the user manual for AGREE II tool (http://www.agreetrust.org/). Ultimately, one reviewer summed all of the scores of the individual items to calculate each domain score using the following formula: (obtained score − minimum possible score/maximum possible score − minimum possible score) × 100%.

After the above steps, we provided an overall assessment of each set of guidelines. A guideline was “strongly recommended for use in practice” if most domains (4 or more) scored above 60%. A guideline was “recommended for use with some modification” if most domains scored between 30% and 60%. “Not recommended for use in practice” implied that most of the domains of the guideline were scored as approximately or below 30%.

## 3. Results

**Figure 1:**
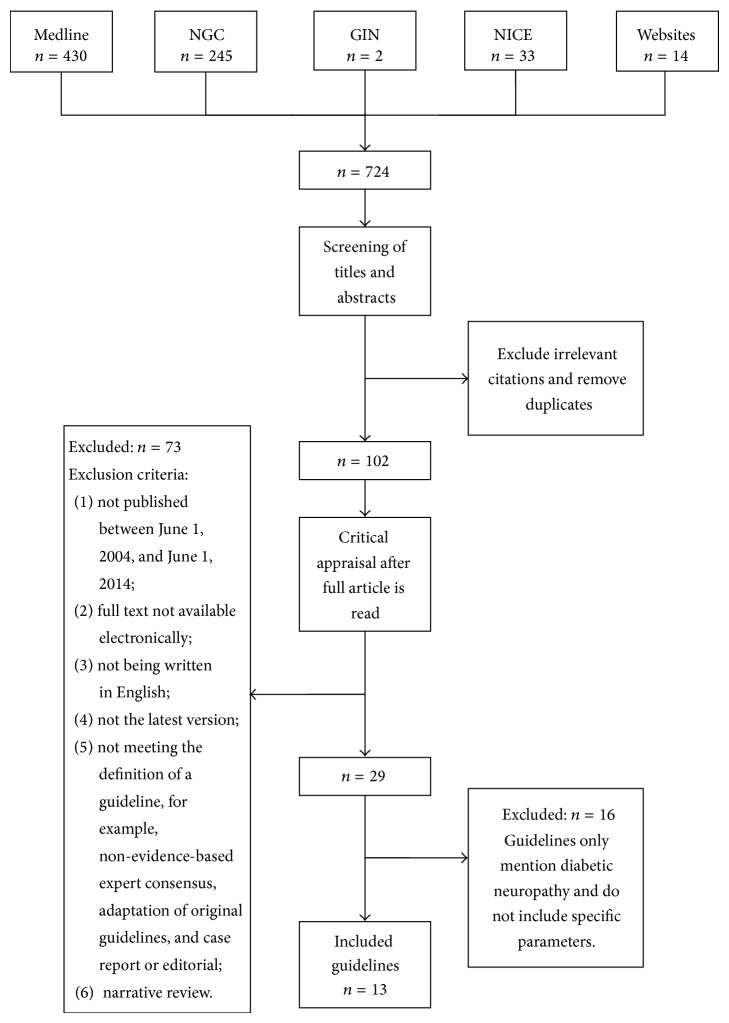
Search Strategies. “Medline” = Medline database; NGC = National Guideline Clearinghouse; GIN = Guidelines International Network; Websites = homepages of international medical societies and institutions or other relevant websites. NICE = National Institute for Health and Clinical Excellence.

A total of 102 guidelines were collected during the primary literature selection process, though many were excluded after applying the inclusion and exclusion criteria. Because specific guidelines for focal and multifocal DN are lacking, we merely evaluated the guidelines for generalized symmetric polyneuropathies. Ultimately, 13 guidelines [3, 11, 15–25] were identified for further evaluation (Figure 1); seven were considered as general guidelines [15–19, 22, 25], and six were considered as specialized guidelines [3, 11, 20, 21, 23, 24]. Further information about the included guidelines is presented in Table 1.

### 3.1. Comparison of the Recommendations for the Management of Diabetic Neuropathy

The recommendations in these guidelines can be divided into three parameters: avoidance of risk factors and complications, treatment based on the pathogenetic mechanisms of DN, and symptom management. The differences and similarities between the guidelines will be discussed for each of these parameters.

The first parameter analyzed was the avoidance of risk factors and complications. The recommendations in this domain were consistent to some extent. Nine guidelines reached unanimous agreement on stable and optimal glycemic control. Providing comprehensive foot care was suggested by eight guidelines. Intensive multifactorial interventions targeting blood pressure, glucose and lipid levels, and other lifestyle factors were considered by five guidelines, whereas six guidelines proposed that patients make lifestyle modifications. Moreover, seven guidelines concurred that patients should receive psychological support (Table 2).

The second parameter focused on treatment based on the pathogenetic mechanisms of DN. The antioxidant alpha-lipoic acid was recommended for pathogenetic treatment by two guidelines, and the guidelines provided by the Statement by American Diabetes Association (SADA) and the Working Group on the Diabetic Foot from the French-Speaking Society of Diabetology (SFD) advised implementing angiotensin-converting enzyme inhibitors to improve microcirculation in neuropathy patients. Nevertheless, the remaining eight guidelines failed to provide any recommendations on this topic (Table 2).

The final parameter aimed at symptom management. Many recommendations were concordant across the guidelines. The fundamental suggestion was the management of painful DN. All guidelines reached a consensus with respect to the use of tricyclic antidepressants, calcium channel *α*2-*δ* ligands, and serotonin noradrenaline reuptake inhibitor drugs for pain relief. Moreover, several guidelines advised patients to utilize certain nonpharmacological interventions. In addition, some guidelines offered explicit first- to fourth-line therapies and drug combinations (Table 3). Furthermore, six guidelines included suggestions for the management of autonomic dysfunction (Table 4).

### 3.2. Quality Assessment

The results of the assessment of the three different aspects of DN management performed using the AGREE II instrument are illustrated in Tables 5–7. In accordance with the calculated statistics, the NICE and Scottish Intercollegiate Guidelines Network (SIGN) guidelines performed sufficiently well and are strongly recommended for use regarding the avoidance of risk factors and complications and symptom management components. The guidelines for neuropathic pain from NICE (PNICE) also perfectly met the criteria of the symptom management component and are strongly recommended for use.

Regarding the avoidance of risk factors and complications parameter, most guidelines received perfect scores for D4 (clarity of presentation), with an average score of 61.9% (Table 5). However, for the pathogenetic mechanism-based treatment parameter, the majority of guidelines did not receive high scores in most of the domains; therefore these guidelines are likely inadequate for use in practice with regard to this component of DN treatment (Table 6). Almost all of the guidelines had relatively poor grades for D5 (applicability) and D6 (editorial independence) for all three parameters.

## 4. Discussion

CPGs are now ubiquitous in almost every aspect of clinical practice and health policy, and it is commonly believed that the proper use of high-quality guidelines can improve patient outcomes. In the modern era, health care practitioners may easily obtain guidelines from web-based guideline databases. To the best of our knowledge, our study is the first comprehensive and detailed evaluation of the quality of international evidence-based guidelines according to three independent aspects of DN treatment. This paper offers an overview of the quality and content of various international guidelines for the management of DN. We evaluated 13 guidelines using AGREE II tool and found that the overall quality of most of the guidelines was relatively moderate, with the exception of the SIGN, NICE, and PNICE guidelines. In general, some key recommendations provided by the included guidelines were discrepant, making it a bit difficult for diabetes clinicians working in busy clinical practices to choose the appropriate course of action.

### 4.1. Quality of the Recommendations for Avoiding Risk Factors and Complications

Our study revealed that most guidelines had identical recommendations concerning this aspect. However, AGREE II (Table 5) scores indicated that none of the included CPGs performed sufficiently well in domain 5 (average score of 19.4%), the applicability domain, implying that the quality of these guidelines was below the expected standards in this domain, thereby limiting the likelihood of their implementation. This relatively low quality makes it more difficult for practitioners to apply these highly consistent recommendations, and as a result, fewer patients will benefit from the guidelines despite their soundness. In addition, two guidelines suggested that patients with diabetes aggressively maintain control of their blood pressure and their blood lipid levels, whereas the other guidelines did not present this recommendation. In fact, a cohort study of the association of vascular risk factors with DN [26, 27] revealed that treating dyslipidemia and hypertension may slow or prevent the progression of DN; however, few interventional clinical trials have addressed this issue. Therefore, further studies should concentrate on establishing high-quality trials or meta-analyses of this issue to bridge the evidence gap.

### 4.2. Quality of the Recommendations regarding Treatment Based on the Pathogenetic Mechanisms of DN

Only four guidelines (SADA, SFD, Toronto Expert Panel on Diabetic Neuropathy (TEPDN), and American Academy of Neurology (AAN)) made any recommendations regarding this aspect. In fact, over the past two decades, many mechanism-based translational medicine investigations and clinical trials have focused on the pathogenetic management of DN. To our surprise, numerous guidelines appeared to fail to mention this aspect, which may indicate that these guidelines may not use systematic methods to search for evidence. Although four guidelines provided explicit recommendations, they unfortunately did not receive high scores using AGREE II tool (average scores of 19.2%, 21.9%, 19.9%, and 18.6%, resp.) (Table 6), suggesting that these recommendations might not be trustworthy and are of very limited practical value. Moreover, our findings suggested that although the recommendations regarding novel topics in these guidelines were generally conservative, there were some differences between the guidelines. For example, the SADA and TEPDN guidelines recommended alpha-lipoic acid for the treatment of DN, whereas the AAN and SFD guidelines advised against its use. Our study demonstrated that these four guideline development panels failed to provide detailed descriptions of how the existing recommendations and evidence were identified and selected, even though providing this piece of information is fundamental for the development of a valid and reliable evidence-based recommendation. This deficiency suggests that these guidelines may include suggestions based on limited evidence, consensus, or expert opinions without the support of clear evidence. In addition, we found that the SADA and SFD guidelines advised neuropathy patients to use additional drugs to improve nerve metabolic disorders or microcirculation. This recommendation might be included for the following reasons. (1) The last update of the SADA guidelines was in the year 2005, longer ago than the other guidelines, and the detailed descriptions of the procedures for updating these guidelines were poor. It is generally recommended that guidelines be updated at least every 3 years, as new evidence is likely to change the recommendations in previous guidelines [28]. These observations highlight the importance of promptly updating a set of guidelines when new clinically relevant evidence becomes available. (2) Local resources are unevenly distributed throughout the world. In fact, disagreement between recommendations is not necessarily a sign of poor guideline quality. Guidelines may disagree because of the value system of the panel that developed them. The best possible care for patients may also vary according to the availability of domestic resources, cost, health priorities, and the social environment. These factors may also influence the recommendations. For example, the SFD guidelines noted that the antioxidative properties of alpha-lipoic acid may improve peripheral neuropathy symptoms but that this product is not available in France. Therefore, the SFD guidelines rejected this drug. Furthermore, the poor scores for editorial independence (D6, average score of 5.5%) offer another explanation for the disagreements between guidelines. The relatively poor performance in this domain could represent true conflicts of interest between funding sources and guideline development panels. It is apparent that the composition of the panel may influence the guidelines and that the resulting recommendations may be vulnerable to the conflicts of interest of the panelists [29].

### 4.3. Quality of the Recommendations regarding Symptom Management

Most guidelines received good scores on this aspect (Table 7). We noted that most guidelines offered detailed recommendations, as well as dosages, for first- to fourth-line drugs and combination therapy. Nevertheless, some distinctions remained, and these inconsistencies may be attributed in part to the relatively low scores in domain 3 (rigor of development) and to the differential interpretation of the same evidence by different guideline developers. For example, our study revealed that even though the SADA and AAN guidelines were based on the same evidence [30], their recommendations for the use of topiramate were opposite. This discrepancy may be caused by the ambiguous evidence on this topic or the fact that the same evidence can be interpreted differently. Clearly, neither the establishment of a consensus nor the use of the same evidence ensures that different guidelines will include identical recommendations.

Regarding the first- to fourth-line drugs, there were discrepancies across most of the guidelines, likely due to the ambiguity of the grading standard. There were rare normative or clear standards for the definition of a first- or second-line drug worldwide, and the specific boundary between first- and second-line drugs is unclear. It is clear that the definitions of first- and second-line therapy should be standardized in further studies.

In regard to combination therapy, it is well known that combination therapy is commonly prescribed for neuropathic pain. Combination therapy may also be a helpful option as a stepwise approach if the initially used drugs insufficiently decrease pain. Combination therapy may also result in greater tolerability [20]. However, few studies have tested the efficacy of combination drug therapies or performed head-to-head treatment comparisons, and studies that include the clinical and cost effectiveness and the tolerability of different drug combinations are rare. Therefore, the current evidence may not be sufficient to warrant any recommendation on combination therapies, and the current suggestions might vary widely, precisely as demonstrated by our study. Without a doubt, recommendations should not be made when the evidence is weak; nevertheless, clinicians still need to make decisions in their daily practice. In fact, some recommendations are validated and reasonable for use in our daily practice, but many recommendations lack credibility because there is limited evidence to support them. Clearly, combination therapies should be further explored, especially head-to-head trials comparing the clinical and cost effectiveness and the tolerability of different drug combinations.

### 4.4. Focal and Multifocal Diabetic Neuropathy

Cranial neuropathy, truncal neuropathy, focal limb neuropathy, proximal motor neuropathy, and chronic inflammatory demyelinating polyneuropathy are not uncommon and are important aspects of DN, which has a serious impact on the quality of life of patients with diabetes [11]. However, during the literature selection process, we found that specific guidelines for these neuropathies are lacking. The absence of such guidelines may be due to a lack of research in this field or to the lack of the use of systematic methods by the guideline development panel to search for evidence. In fact, general guidelines and recommendations appear to be unsuitable for focal and multifocal neuropathies. Undoubtedly, further studies should concentrate on searching for new evidence regarding the treatment of focal and multifocal neuropathies, and subsequent guideline development panels should formulate guidelines that could be applied to focal and multifocal DN so that more patients can benefit from these guidelines and ultimately achieve improved outcomes.

In conclusion, the results of this study indicate that despite the general acceptance of the approach and quality criteria related to the development of evidence-based guidelines, different guidelines may produce conflicting conclusions and differing recommendations for similar clinical situations. Some discrepancies are valid and reasonable, but others may not. Therefore, the quality of most guidelines for the treatment of DN should be improved. Clinical practice guidelines should be developed according to AGREE II criteria to support good clinical practice and effective patient care. High-quality trials and meta-analyses should be used during the guideline development process, and different societies and countries should collaborate in guideline development to generate consensus recommendations for clinical practice. Further studies should concentrate on establishing internationally accepted and evidence-based guidelines that could be used for clinical decision making regarding the treatment of DN to enhance patient care and to improve patient outcomes.

## Figures and Tables

**Table 1:** Description of the guidelines included in this study.

	Organization	Short name	Relevant countries	Last update
Standards of Medical Care in Diabetes-2014	American Diabetes Association	ADA	United States	2014

Management of Type 2 Diabetes Mellitus	University of Michigan Health System	UMHS	United States	2014

Neuropathic Pain: Pharmacological Management	National Institute for Health and Clinical Excellence	PNICE^*^	England and Wales	2013

Global Guideline for Type 2 Diabetes	International Diabetes Federation	IDF	International	2012

American Association of Clinical Endocrinologists Medical Guidelines for Clinical Practice for Developing a Diabetes Mellitus Comprehensive Care Plan	The American Association of Clinical Endocrinologists	AACE	United States	2011

Guidance on the Management of Type 2 Diabetes	New Zealand Guidelines Group	NZGG	New Zealand	2011

Evidence-Based Guideline: Treatment of Painful Diabetic Neuropathy	American Academy of Neurology	AAN	United States	2011

Painful Diabetic Peripheral Neuropathy Consensus: Recommendations on Diagnosis, Assessment, and Management	Toronto Expert Panel on Diabetic Neuropathy	TEPDN	Canada	2011

Painful Diabetic Neuropathy: Diagnosis and Management	Working Group on the Diabetic Foot from the French-Speaking Society of Diabetology	SFD	France	2011

Management of Diabetes: A National Clinical Guideline	Scottish Intercollegiate Guidelines Network	SIGN	Scotland	2010

EFNS Guidelines on the Pharmacological Treatment of Neuropathic Pain: 2010 Revision	European Federation of Neurological Society	EFNS	Europe	2010

Type 2 Diabetes: The Management of Type 2 Diabetes	National Institute for Health and Clinical Excellence	NICE	England and Wales	2009

Diabetic Neuropathies: A Statement by the American Diabetes Association	American Diabetes Association	SADA	United States	2005

PNICE^*^ indicates the guidelines for neuropathic pain from the National Institute for Health and Clinical Excellence.

**Table 2:** Recommendations for minimizing risk factors and complications and treatment-based pathogenetic mechanisms of diabetic neuropathy.

			ADA	UMHS	PNICE	IDF	AACE	NZGG	AAN	TEPDN	SFD	SIGN	EFNS	NICE	SADA
Risk factors and complications	Glycemic factor	Stable and optimal glycemic control	●	●	—	●	●	●	—	●	○	●	—	●	●
	Nonglycemic factor	Blood pressure control	—	—	—	—	●	—	—	—	—	—	—	—	●
		Blood lipid level control	—	—	—	—	●	—	—	—	—	—	—	—	●
		Lifestyle modification	—	●	—	—	●	—	—	●	—	●	—	●	●
	Multifactorial intervention		●	—	—	●	—	●		●	—	●	—	—	—
	Foot care	Foot self-care education	●	●	—	●	●	●	—	—	—	●	—	●	●
		Regular comprehensive foot exam	●	●	—	●	●	●	—	—	—	●	—	●	●
		Referral to multidisciplinary foot-care team	●	●	—	●	—	●	—	—	—	●	—	●	●
	Psychological support^*^		●	●	●	●	—	●	—	—	—	●	—	●	—
Pathogenetic mechanisms	Reducing oxidative stress	Alpha-lipoic acid	—	—	—	—	—	—	○	●	○	—	—	—	●
	Improving metabolic disorder	Epalrestat	—	—	—	—	—	—	—	—	—	—	—	—	●
		Fidarestat	—	—	—	—	—	—	—	—	—	—	—	—	●
		Ranirestat	—	—	—	—	—	—	—	—	—	—	—	—	●
	Improving microcirculation	ACE inhibitors	—	—	—	—	—	—	—	—	●	—	—	—	●
		Prostaglandin analogs	—	—	—	—	—	—	—	—	—	—	—	—	●

● indicates being recommended; ○ indicates being not recommended; — indicates being not mentioned.

Psychological support^*^ indicates assessment of psychological stress or antidepression therapy.

ACE indicates angiotensin-converting enzyme. IDF indicates the International Diabetes Federation. NICE indicates the National Institute for Health and Clinical Excellence. SIGN indicates the Scottish Intercollegiate Guidelines Network. AACE indicates the American Association of Clinical Endocrinologists. NZGG indicates the New Zealand Guidelines Group. AAN indicates the American Academy of Neurology. TEPDN indicates the Toronto Expert Panel on Diabetic Neuropathy. SADA indicates Statement by the American Diabetes Association. PNICE indicates the guidelines for neuropathic pain from the National Institute for Health and Clinical Excellence. ADA indicates the American Diabetes Association. SFD indicates the Working Group on the Diabetic Foot from the French-Speaking Society of Diabetology. EFNS indicates the European Federation of Neurological Societies. UMHS indicates the University of Michigan Health System.

**Table 3:** Recommendations regarding symptom management for painful neuropathy.

	Drug	ADA	UMHS	PNICE	IDF	AACE	NZGG	AAN	TEPDN	SFD	SIGN^*^	EFNS	NICE^*^	SADA
Antipyretic analgesics (AA)	Acetaminophen	—	●	—	—	—	●**1**	—	—	○	—	●	—	—
Tricyclic antidepressants (TCA)	Amitriptyline	●	—	●**1**	●**1**	●**1**	●**2**	●	●**1**	●**1**	●	●**1**	●**1**	●**1**
	Nortriptyline	—	●**1**	—	—	—	—	○	○	●	—	—	—	—
	Imipramine	—	—	—	—	—	●**2**	○	●**1**	●	●	—	●**1**	●**1**
	Desipramine	—	—	—	—	—	●**2**	○	○	●	●	—	●**1**	—
	Clomipramine	—	—	○	—	—	—	—	—	●**1**	—	●	—	—
Antiepileptics (A)	Pregabalin	●	●**1**	●**1**	●**2**	●**2**	●**3**	●	●**1**	●**1**	●	●**1**	●	●
	Gabapentin	●	●**1**	●**1**	●**2**	●**2**	●**3**	●	●**1**	●**1**	●	●**1**	●	●**2**
Other antiepileptics (OA)	Carbamazepine	—	●	—	—	—	●**4**	—	●	○	●	○	○	●
	Oxcarbazepine	—	—	○	—	●**3**	—	○	—	○	—	○	○	—
	Valproate	●	●	—	—	—	—	●	—	○	—	○	—	—
	Topiramate	—	—	○	—	—	—	○	●	○	—	○	—	●
	Lamotrigine	—	—	○	—	—	—	○	—	○	—	○	—	—
SNRIs (S)	Duloxetine	●	●**1**	●**1**	●**2**	●**1**	●	●	●**1**	●**1**	●	●**1**	●	●
	Venlafaxine	●	●	○	—	—	—	●	●	—	●	●**1**	—	—
Opiates (O)	Tramadol	●	●	●**2**	●**3**	●**3**	—	●	●	●**2**	—	●**2**	●**3**	●**3**
	Morphine	●	—	○	—	—	—	●	●	●	—	●	●**3**	—
	Oxycodone	●	—	—	●**3**	—	—	●	●	●**2**	—	●	●**3**	●**3**
Nonpharmacological interventions	Topical treatment	—	●	●	—	●**3**	●	●	●	●	○	○	—	●
	Physical therapy	—	●	—	—	—	—	●	○	●	○	—	—	●
Combination therapies	TCA + S	—	—	○	—	—	—	—	●	—	—	—	●	●**2**
	TCA + A	—	—	○	—	—	—	—	●	●**2**	—	—	●	—
	S + A	—	—	○	—	—	—	—	●	—	—	—	—	—
	O + TCA + S	—	—	○	—	—	—	—	●	—	—	—	●	—
	O + TCA + A	—	—	○	—	—	—	—	●	—	—	—	●	—
	O + S + A	—	—	○	—	—	—	—	●	—	—	—	—	—
	O + A	—	—	○	—	—	—	—	—	●**2**	●	●	—	—
	O + AA	—	●	○	—	—	—	—	—	—	—	●	—	—
	O + TCA	—	—	○	—	—	—	—	—	●**2**	—	●	—	—
Referral to pain control team		●**4**	—	—	●	●**4**	—	—	—	—	—	—	—	●**4**

● indicates being recommended; ○ indicates being not recommended; — indicates being not mentioned.

●**1** indicates being recommended for first-line therapy; ●**2** indicates being recommended for second-line therapy;

●**3** indicates being recommended for third-line therapy; ●**4** indicates being recommended for fourth-line therapy.

SNRI indicates serotonin noradrenaline reuptake inhibitor.

^*^The NICE and SIGN guidelines mentioned opiates, but no specific drugs were suggested.

**Table 4:** Recommendations for autonomic dysfunction management.

	Symptom	Management	ADA	UMHS	PNICE	IDF	AACE	NZGG	AAN	TEPDN	SFD	SIGN	EFNS	NICE	SADA
Cardiac system	Exercise intolerance		—	—	—	—	●	—	—	●	—	—	—	—	●
	Orthostatic hypotension		●	—	—	—	●	—	—	●	—	—	—	—	●
Gastrointestinal system	Gastroparesis	Metoclopramide	●	—	—	●	●	—	—	—	—	—	—	●	●
		Domperidone	—	—	—	●	●	—	—	—	—	—	—	●	●
		Erythromycin	●	—	—	—	●	—	—	—	—	—	—	●	●
	Constipation		—	—	—	—	●	—	—	—	—	—	—	—	●
	Diarrhea		—	—	—	—	●	—	—	—	—	—	—	●	●
	Abdominal discomfort		—	—	—	—	●	—	—	—	—	—	—	—	●
Urogenital system	Erectile dysfunction	PDE5 inhibitor	●	—	—	●	●	—	—	—	—	—	—	●	●
		Prostaglandins	●	—	—	—	●	—	—	—	—	—	—	—	●
		Vacuum device	●	—	—	—	●	—	—	—	—	—	—	—	●
		Surgery	●	—	—	—	●	—	—	—	—	—	—	●	●
		Psychological counseling	—	—	—	●	●	—	—	—	—	—	—	●	●
	Vaginal dryness	Lubricant	—	—	—	—	●	—	—	—	—	—	—	—	●
	Bladder dysfunction		—	—	—	—	●	—	—	—	—	—	—	—	●
Sudomotor dysfunction			—	—	—	—	●	—	—	—	—	—	—	●	●
Pupillomotor and visceral dysfunction			—	—	—	—	●	—	—	—	—	—	—	—	●

● indicates recommended; — indicates not mentioned.

IDF indicates the International Diabetes Federation. NICE indicates the National Institute for Health and Clinical Excellence. SIGN indicates the Scottish Intercollegiate Guidelines Network. AACE indicates the American Association of Clinical Endocrinologists. NZGG indicates the New Zealand Guidelines Group. AAN indicates the American Academy of Neurology. TEPDN indicates the Toronto Expert Panel on Diabetic Neuropathy. SADA indicates Statement by the American Diabetes Association. PNICE indicates the guidelines for neuropathic pain from the National Institute for Health and Clinical Excellence. ADA indicates the American Diabetes Association. SFD indicates the Working Group on the Diabetic Foot from the French-Speaking Society of Diabetology. EFNS indicates the European Federation of Neurological Societies. UMHS indicates the University of Michigan Health System.

**Table 5:** Results of the assessment of recommendations regarding the avoidance of risk factors and complications using the AGREE II instrument (domain scores in %).

	D1	D2	D3	D4	D5	D6	Ave	Overall assessment
ADA	55.6	50.0	35.4	100.0	0.0	50.0	48.5	M
UMHS	50.0	33.3	25.0	44.4	4.2	50.0	34.5	M
AACE	50.0	33.3	31.3	44.4	0.0	16.7	29.3	M
PNICE	0.0	0.0	0.0	0.0	0.0	0.0	0.0	N
IDF	50.0	22.2	43.8	94.4	54.2	33.3	49.7	M
NZGG	77.8	55.6	33.3	77.8	45.8	50.0	56.7	M
AAN	0.0	0.0	0.0	0.0	0.0	0.0	0.0	N
TEPDN	33.3	27.8	68.8	51.4	11.1	23.8	36.0	N
SFD	0.0	27.8	14.6	38.9	25.0	25.0	21.9	N
SIGN	88.9	88.9	81.3	100.0	95.8	33.3	81.4	SR
EFNS	0.0	0.0	0.0	0.0	0.0	0.0	0.0	N
NICE	72.2	77.8	87.5	100.0	16.7	58.3	68.8	SR
SADA	55.6	0.0	3.5	51.4	0.0	0.0	18.4	N
Ave	41.0	32.1	32.7	54.1	19.4	26.2		

Ave indicates average. SR indicates being strongly recommended for use in clinical practice. M indicates being recommended for use in clinical practice with some modifications. N indicates being not recommended for use in clinical practice.

D1 (domain 1) indicates scope and purpose; D2 (domain 2) indicates stakeholder involvement; D3 (domain 3) indicates rigor of development; D4 (domain 4) indicates clarity of presentation; D5 (domain 5) indicates applicability; D6 (domain 6) indicates editorial independence.

**Table 6:** Results of the assessment of the recommendations regarding treatment based on pathogenetic mechanisms using the AGREE II instrument (domain scores in %).

	D1	D2	D3	D4	D5	D6	Ave	Overall assessment
ADA	0.0	0.0	0.0	0.0	0.0	0.0	0.0	N
UMHS	0.0	0.0	0.0	0.0	0.0	0.0	0.0	N
AACE	50.0	33.3	0.0	0.0	0.0	16.7	16.7	N
PNICE	0.0	0.0	0.0	0.0	0.0	0.0	0.0	N
IDF	0.0	0.0	0.0	0.0	0.0	0.0	0.0	N
NZGG	0.0	0.0	0.0	0.0	0.0	0.0	0.0	N
AAN	55.6	22.2	0.0	21.0	8.8	4.2	18.6	N
TEPDN	33.3	27.8	0.0	33.3	0.0	25.0	19.9	N
SFD	0.0	27.8	14.6	38.9	25.0	25.0	21.9	N
SIGN	0.0	0.0	0.0	0.0	0.0	0.0	0.0	N
EFNS	0.0	0.0	0.0	0.0	0.0	0.0	0.0	N
NICE	0.0	0.0	0.0	0.0	0.0	0.0	0.0	N
SADA	55.6	0.0	4.2	51.4	4.2	0.0	19.2	N
Ave	15.0	8.5	1.4	11.1	2.9	5.5		

Ave indicates average. SR indicates being strongly recommended for use in clinical practice. M indicates being recommended for use in clinical practice with some modifications. N indicates being not recommended for use in clinical practice.

D1 (domain 1) indicates scope and purpose; D2 (domain 2) indicates stakeholder involvement; D3 (domain 3) indicates rigor of development; D4 (domain 4) indicates clarity of presentation; D5 (domain 5) indicates applicability; D6 (domain 6) indicates editorial independence.

**Table 7:** Results of the assessment of the symptom management recommendations using the AGREE II instrument (domain scores in %).

	D1	D2	D3	D4	D5	D6	Ave	Overall assessment
ADA	55.6	50.0	31.3	61.1	0.0	50.0	41.3	M
UMHS	50.0	33.3	25.0	44.4	4.2	50.0	34.5	M
AACE	50.0	33.3	31.3	44.4	0.0	16.7	29.3	M
PNICE	100.0	61.1	75.0	100.0	37.5	50.0	70.6	SR
IDF	50.0	22.2	39.6	88.9	54.2	33.3	48.0	M
NZGG	77.8	55.6	33.3	94.4	45.8	50.0	59.5	M
AAN	55.6	22.2	68.8	88.9	8.3	50.0	49.0	M
TEPDN	33.3	27.8	6.3	50.0	0.0	25.0	23.7	N
SFD	0.0	27.8	14.6	38.9	25.0	25.0	21.9	N
SIGN	88.9	88.9	81.3	100.0	95.8	33.3	81.4	SR
EFNS	33.3	38.9	35.4	61.1	12.5	25.0	34.4	M
NICE	72.2	77.8	87.5	100.0	16.7	58.3	68.8	SR
SADA	55.6	0.0	4.2	44.4	4.2	0.0	18.1	N
Ave	55.6	41.5	41.0	70.5	23.4	35.9		

Ave indicates average. SR indicates being strongly recommended for use in clinical practice. M indicates being recommended for use in clinical practice with some modifications. N indicates being not recommended for use in clinical practice.

D1 (domain 1) indicates scope and purpose; D2 (domain 2) indicates stakeholder involvement; D3 (domain 3) indicates rigor of development; D4 (domain 4) indicates clarity of presentation; D5 (domain 5) indicates applicability; D6 (domain 6) indicates editorial independence.
